# Combinations of *Alchornea cordifolia, Cassytha filiformis* and *Pterocarpus santalinoides* in diarrhoegenic bacterial infections

**DOI:** 10.1186/s13104-019-4687-0

**Published:** 2019-10-07

**Authors:** Angus Nnamdi Oli, Monday Obaji, Ifeoma Bessie Enweani

**Affiliations:** 1 Department of Pharmaceutical Microbiology and Biotechnology, Faculty of Pharmaceutical Sciences, Nnamdi Azikiwe University, Awka, Nigeria; 2 Department of Medical Laboratory Science, Faculty of Health Science and Technology, College of Health Sciences, Nnamdi Azikiwe University, Nnewi, Nigeria

**Keywords:** *Cassytha filiformis*, *Pterocarpus santalinoides*, *Alchornea cordifolia*, *Escherichia coli*, *Shigellae dysenteriae*, *Salmonella typhi*, *Staphylococcus aureus*

## Abstract

**Objectives:**

This study examines the rationale, if any, behind combining the extracts from the fruits of *Alchornea cordifolia* and *Pterocarpus santalinoides* and aerial parts of *Cassytha filiformis* in the traditional treatment of diarrhoegenic bacterial infections.

**Results:**

Four diarrhoegenic bacterial isolates: *Salmonella typhi*, *Shigellae dysenteriae*, *Escherichia coli* and *Staphylococcus aureus* were used and their antibiotic susceptibility screening showed that they were multi-antibiotic resistant. The extracts exhibited activity against all the test isolates with minimum inhibitory concentration values ranging from 3.125 to 12.5 mg/mL. From the checkerboard assay, the fractional inhibitory concentration indices showed that *C. filiformis* has antagonistic and indifference activities in combination with either *P. santalinoides* or *A. cordifolia.* This showed that the combination of extracts from the fruits of *A. cordifolia* and *P. santalinoides* and aerial parts of *C. filiformis* is counterproductive and invalidates any claim for positive results in the management of diarrhoegenic bacterial infections.

## Introduction

Plants are age-long sources of medicine to mankind especially in developing world. Besides serving as source of food, plants have been a potent weapon for combating diseases. Both traditional healers and pharmaceutical researchers have continuously looked up to plants to provide cure to both infectious and non-infectious diseases. For instance, the root, stem, leaves and fruits of *Cassytha filiformis* are widely used for the treatment of ulcer, cancer, conjunctivitis, diarrhoea, erectile dysfunction, post-partum haemorrhage, GIT infection and urinary tract infections [1–3]. Plants have been particularly helpful in the fight against infectious diseases because of the evolution of microorganisms with extraordinary ability to render conventional antibiotics relatively ineffective.

The leaves of *Pterocarpus santalinoides* are used for treating diarrhoea of bacterial and non-bacterial origin [4], have hypolipidemic effect [5] and are rich in phytochemical compounds such as flavonoid and alkaloid as well as being very good sources of vitamins [6]. Eze et al. [7] have also reported that various parts of *Pteropus santalinoides* are utilised in folk medicine for the treatment of malaria.

Decoctions of *Alchornea cordifolia* fruit, leaf or stem bark are utilised for the treatment of venereal diseases, cough, and impotency [8]. They are also utilised by traditional medicine practitioners for the treatment of diarrhoea [8, 9].

Diarrhoea is one of the leading causes of death among children below 5 years [10, 11] and diarrhoeal diseases are especially dangerous among the immune-incompetent populations [12, 13]. It was estimated that, globally, over 500,000 children below 5 years died of acute diarrhoea in 2015 only [11, 14].

All over the developing world, especially in rural communities, diarrhoea accounts for most incidences of child morbidity and mortality [8]. Diarrhoea may be caused by both infectious microorganism and non-pathogenic agents. Enterotoxigenic *E. coli* and *Shigellae* spp. are amongst the leading causes of diarrhoea globally [15, 16]. Other bacteria implicated in diarrhoea condition are *Staphylococcus aureus* and *Salmonella typhi*. *S. aureus* produces enterotoxin, which causes inflammation of the ileum and diarrhoea [17, 18].

Since the discovery of antibiotics, they have been widely utilised for the treatment of infectious diseases because they have proven to be efficacious against dangerous pathogens. However, with the increasing incidences of antibiotic resistance amongst clinically important pathogens, clinicians may no longer completely depend on antibiotics for the treatment of all infectious diseases. Several studies have presented plants as alternative to conventional drugs for the treatment of infectious diseases [19–21]. Beside their efficacy against resistant strains, plants are acclaimed to have phyto compounds that have very little or no adverse effects compared to conventional drugs [22, 23]. Traditional healers have always combined several herbs in a bid to harness the therapeutic effects of more than one plant to cure diseases. Also, drug combinations have been particularly helpful in the fight against infectious diseases because of the evolution of pathogens with extraordinary abilities to render conventional antibiotics relatively ineffective. This study seeks to evaluate the in vitro interactions of the methanolic herbal extracts from the fruits of *A. cordifolia*, *P. santalinoides* and aerial parts of *C. filiformis*. The result is expected to either justify, or otherwise invalidate, the local use of the concoctions containing these plants’ parts in the management of childhood diarrhoea associated with the tested bacteria.

## Main text

### Test isolates

All the isolates used for this study were obtained from previous stocks deposited in the laboratory of the Department of Pharmaceutical Microbiology and Biotechnology, Faculty of Pharmaceutical Sciences, Nnamdi Azikiwe University, Awka. Isolates were originally from diarrhoeic stool of children below 5 years of age. Isolates were subcultured on Mannitol salt agar, Salmonella-Shigellae agar and MacConkey agar respectively and appropriate biochemical tests undertaken as described by Salami and Georgia [24].

Plant samples collection, preparation and extraction: the fruits of *A. cordifolia*, *P. santalinoides* and aerial parts of *C. filiformis* were harvested from a farmland at Nnewi, Nigeria. They were authenticated by a taxonomist: Okeke Philomena N of the Department of Botany, Nnamdi Azikiwe University, Awka and deposited in the departmental herbarium with the voucher codes: *A. cordifolia*—78D; *P. santalinoides*—52A; *C. filiformis*—124A. The samples were washed with clean water, air-dried under shade and pulverized. The methanolic extraction of the pulverized samples was carried out using a method described by Basri and Fan [25] with little modification. A 500 g of the pulverized samples were immersed in 2.5 L of methanol for 24 h with shaking. The resultant mixtures were filtered using muslin cloth, then with Whatman no. 1 filter. The filtrates were concentrated using rotary evaporator and further concentrated by heating in water bath. The crude extracts were stored in a refrigerator at temperature 4–8 °C until ready for use.

### Antibiotic susceptibility assay

Test isolates were challenged with several antibiotics using disc diffusion method as described by Cheesbrough [26]. The test inocula were prepared with 24 h broth cultures and then adjusted with physiological saline until they are equivalent to McFarland 0.5 turbidity standard. The standardised inocula were applied on the surface of solidified sterile Mueller–Hinton agar in a Petri dish and allowed to stand. Commercial antibiotic discs were gently placed on the agar using sterilised forceps. The cultures were then incubated at 37 °C for 24 h. The zones of inhibition were measured using transparent plastic rule.

Determination of minimum inhibitory concentration (MIC): the MICs of the extracts were determined using agar well diffusion assay as described by Ghamba et al. [27]. The extracts were prepared by dissolving 200 mg of the extracts in 2 mL of DMSO to obtain a concentration of 100 mg/mL. Dilutions of 50, 25, 12.5, 6.25 and 3.125 mg/mL were prepared from the 100 mg/mL stock solutions of the six extracts. Sterilised cork borer (6 mm in diameter) was used to make holes on solidified Muller Hinton agar containing the standardised pure culture of the test isolate. Each concentration of the extracts was carefully introduced into the holes and allowed to stand for 1 h before incubating at 37 °C for 24 h. The least concentration (measured in milligram) with zone of inhibition (measured in millimetre) is considered the MIC of the extract.

Combined antimicrobial activities of the extracts (Checkerboard assay): The antimicrobial activities of the extracts in combination were determined using checkerboard assay described by Aiyegoro et al. [28]. The concentrations of the extracts (2 × MIC of individual significant activity against test isolates) were prepared according to a continuous variation checkerboard technique using the ratio 0:10, 1:9, 2:8, 3:7, 4:6, 5:5, 6:4, 7:3, 8:2, 9:1, 10:0. The resulting solutions of these combined extracts were further diluted in 2 fold dilution process to 5—serial dilutions to obtain the final concentrations used for the Checkerboard assay. Sterile molten Mueller–Hinton agar was inoculated with 0.1 mL fresh cultures of test isolates and various concentrations of the combined plant extracts. The procedure was carried out in triplicates. The FIC of all ratios of the combined extracts were determined and the FIC value for each extract was calculated using the formula:

$${\text{FIC}}_{\text{A}} = \frac{Conc\;of\;A\;in\;MIC\;A + B}{MIC\;of\;A\;alone}$$

$${\text{FIC}}_{\text{B}} = \frac{Conc\;of\;B\;in\;MIC\;A + B}{MIC\;of\;B\;alone}$$

${\text{FIC}}\;{\text{index}} = {\text{FIC}}_{\text{A}} + {\text{FIC}}_{\text{B}}$. The interpretations of FIC index according to Aiyegoro et al. [28] is as follow: FIC index < 1.0 means Synergism, = 1 means additivity, > 1 but less than 2 means indifference while ≥ 2 means antagonism.

## Results

The antibiotic susceptibility assay confirmed the isolates to show multi-antibiotic resistant character (Additional file 1: Table S1), being resistant to more than two classes of antibiotic.

The extracts at various concentrations exerted antibacterial activity against all the test isolates. The least concentration that inhibited the growth of test isolates within 24 h was considered as the MIC. All the extracts have MIC ranges from 3.125 to 12.5 mg/mL (Table 1).

**Table 1:** Minimum inhibitory concentration of the crude extracts and controls on test isolates

Test isolates	*Staphylococcus aureus*	*Salmonella* spp.	*Shigellae dysenteriae*	*Escherichia coli*
Herbal extracts (mg/mL)				
*Alchornea cordifolia*	12.5	6.25	6.25	12.5
*Cassytha filiformis*	6.25	3.125	6.25	6.25
*Pterocarpus santalinoides*	12.5	12.5	6.25	12.5
Metronidazole 50 µg	5.00	6.00	4.00	5.00
Tetracycline 30 µg	9.00	9.00	9.00	8.00
DMSO (2.5% v/v)	0.00	0.00	0.00	0.00

Metronidazole 50 µg and Tetracycline 30 µg were the positive controls while DMSO (2.5% v/v) was the negative control

Tables 2 and 3 showed the fractional inhibitory concentrations of *C. filiformis* in combination with *A. cordifolia* and *P. santalinoides* respectively. The effect of the combinations are generally antagonistic or indifference. *C. filiformis* in combination with *A. cordifolia* against all the test isolates have FIC indices ranging from 1.04 to 2.84 while *C. filiformis* in combination with *P. santalinoides* have FIC indices ranging from 1.00 to 2.72 against all the test isolates.

**Table 2:** Effects of the crude extracts of *Alchornea cordifolia* and *Cassytha filiformis* on test isolates

*AC: CF*	Test isolates	FIC	Interpretation of results
10: 0	*S. aureus* *Salmonella* spp. *S. dysenteriae* *E. coli*	–	–
9: 1	*S. aureus* *Salmonella* spp. *S. dysenteriae* *E. coli*	1.52 1.21 1.42 1.45	Indifference > 1 Indifference > 1 Indifference > 1 Antagonism > 2
8: 2	*S. aureus* *Salmonella* spp. *S. dysenteriae* *E. coli*	2.34 1.56 1.61 2.50	Antagonism > 2 Indifference > 1 Indifference > 1 Antagonism > 2
7:3	*S. aureus* *Salmonella* spp. *S. dysenteriae* *E. coli*	1.63 2.84 2.02 2.62	Indifference > 1 Antagonism > 2 Antagonism > 2 Antagonism > 2
6: 4	*S. aureus* *Salmonella* spp. *S. dysenteriae* *E. coli*	2.03 2.42 1.23 2.08	Antagonism > 2 Antagonism > 2 Indifference > 1 Antagonism > 2
5: 5	*S. aureus* *Salmonella* spp. *S. dysenteriae* *E. coli*	1.84 2.72 2.09 2.71	Indifference > 1 Antagonism > 2 Antagonism > 2 Antagonism > 2
4: 6	*S. aureus* *Salmonella* spp. *S. dysenteriae* *E. coli*	1.04 1.22 1.54 2.32	Indifference > 1 Indifference > 1 Indifference > 1 Antagonism > 2
3: 7	*S. aureus* *Salmonella* spp. *S. dysenteriae* *E. coli*	2.34 2.05 2.11 1.41	Antagonism > 2 Antagonism > 2 Antagonism > 2 Indifference > 1
2: 8	*S. aureus* *Salmonella* spp. *S. dysenteriae* *E. coli*	2.83 1.83 1.26 1.44	Antagonism > 2 Indifference > 1 Indifference > 1 Indifference > 1
1: 9	*S. aureus* *Salmonella* spp. *S. dysenteriae* *E. coli*	1.43 2.42 1.14 1.32	Indifference > 1 Antagonism > 2 Indifference > 1 Indifference > 1
0: 10	*S. aureus* *Salmonella* spp. *S. dysenteriae* *E. coli*	–	–
Metronidazole Tetracycline (positive control)	*S. aureus* *Salmonella* spp. *S. dysenteriae* *E. coli*	0.82 0.81 0.77 0.67	Synergism Synergism Synergism Synergism
DMSO (2.5% v/v) (negative control)	*S. aureus* *Salmonella* spp. *S. dysenteriae* *E. coli*	0.00 0.00 0.00 0.00	No activity No activity No activity No activity

*AC* methanolic *Alchornea cordifolia* extract, *CF* methanolic *Cassytha filiformis* extract

**Table 3:** Effects of the crude extracts of *Cassytha filiformis* and *Pterocarpus santalinoides* on test isolates

*CF: PS*	Test isolates	FIC	Interpretation of results
10: 0	*S. aureus* *Salmonella* Spp. *S. dysenteriae* *E. coli*	–	–
9: 1	*S. aureus* *Salmonella typhi* *S. dysenteriae* *E. coli*	1.40 1.86 1.17 1.00	Indifference > 1 Indifference > 1 Indifference > 1 Additive = 1
8: 2	*S. aureus* *Salmonella typhi* *S. dysenteriae* *E. coli*	2.31 2.12 2.18 1.16	Antagonism > 2 Antagonism > 2 Antagonism > 2 Indifference > 1
7:3	*S. aureus* *Salmonella typhi* *S. dysenteriae* *E. coli*	2.22 1.67 2.72 2.05	Antagonism > 2 Indifference > 1 Antagonism > 2 Antagonism > 2
6: 4	*S. aureus* *Salmonella typhi* *S. dysenteriae* *E. coli*	1.25 1.32 1.64 1.49	Indifference > 1 Indifference > 1 Indifference > 1 Indifference > 1
5: 5	*S. aureus* *Salmonella typhi* *S. dysenteriae* *E. coli*	1.65 1.47 1.18 1.43	Indifference > 1 Indifference > 1 Indifference > 1 Indifference > 1
4: 6	*S. aureus* *Salmonella typhi* *S. dysenteriae* *E. coli*	1.87 2.09 1.63 1.38	Indifference > 1 Indifference > 1 Indifference > 1 Indifference > 1
3: 7	*S. aureus* *Salmonella typhi* *S. dysenteriae* *E. coli*	2.01 2.03 2.53 2.15	Antagonism > 2 Antagonism > 2 Antagonism > 2 Antagonism > 2
2: 8	*S. aureus* *Salmonella typhi* *S. dysenteriae* *E. coli*	2.14 1.92 1.43 1.88	Antagonism > 2 Indifference > 1 Indifference > 1 Indifference > 1
1: 9	*S. aureus* *Salmonella typhi* *S. dysenteriae* *E. coli*	1.52 1.44 1.08 1.62	Indifference > 1 Indifference > 1 Indifference > 1 Indifference > 1
0: 10	*S. aureus* *Salmonella typhi* *S. dysenteriae* *E. coli*	–	–
Metronidazole Tetracycline (positive control)	*S. aureus* *Salmonella* spp. *S. dysenteriae* *E. coli*	0.82 0.81 0.77 0.67	Synergism Synergism Synergism Synergism
DMSO (2.5% v/v) (negative control)	*S. aureus* *Salmonella* spp. *S. dysenteriae* *E. coli*	0.00 0.00 0.00 0.00	No activity No activity No activity No activity

*CF* methanolic *Cassytha filiformis* extract, *PS* methanolic *Pterocarpus santalinoides* extract

## Discussion

Resistance to conventional antibiotics is the biggest hurdle to overcome in the treatment and eradication of infectious diseases. As the resistant strains spread across vulnerable populations (immunocompromised patients and children), finding other antibiotics that are both efficacious and have minimal adverse effects becomes more challenging. The isolates were resistant to more than 2 antibiotic classes, which is in line with the work of Usha et al. [29] that concluded that most bacteria of clinical importance are resistant to one or more antibiotics.

Herbal medicine have been considered as alternative to treating both infectious and non-infectious diseases because of their proven efficacy in traditional medicine practice [30–32]. All the methanolic extracts used for this study showed significant activities against all the test isolates even at low concentrations. However, when two or more of the herbal extracts were combined, the interaction results showed indifference or antagonism. It may be opined from this study that the bioactive principles in one of these extracts either suppresses the effects of the others or produced an outright cancellation of effect at the combination ratios. Similar negative results were also obtained when Adonu et al. [1] evaluated the effects of *C. filiformis* and *Cleistopholis patens* against *Pseudomonas aeruginosa* and *Escherichia coli* [2]. Drugs interactions showing indifferent or autonomy means that the combination is equipotent with the most potent of the drugs used alone while antagonism occurs when one therapeutic agent cancels out or just weakens the effect of another therapeutic agent(s).

The essence of drug combinations is to achieve a higher therapeutic effect than when one drug is used singly. This was not achieved when *C. filiformis* was combined with either *A. cordifolia* or *P. santalinoides*. The usefulness of combined therapeutic agents had been proven in several studies [33, 34]. For instance, the triple antibiotic paste (containing equal amounts of minocycline, ciprofloxacin, and metronidazole) is very effective in eliminating wide range of bacteria causing teeth decay [35]. Combination of tetracycline and metronidazole was found to be effective against multi-drug resistant diarrhoegenic bacteria [36].

Polyherbal medication interactions had been reported to cause a wide range of effects ranging from useful to untoward effects [37–41]. Possible reasons for the harmful effects may include lack of standardization [42], lack of observance of Good Manufacturing Practice and lack of adequate studies to establish efficacy and combinatorial ratios before marketing of the products.

In conclusion, whereas the use of herbal medicine for the treatment of infectious diseases is gaining global acceptance, indiscriminate combination of various plants extracts is strongly discouraged. This study has shown that combining various plants’ extracts do not necessarily yield a higher therapeutic effect. Therefore, there should be scientifically established bases before two or more plant parts are combined for any therapeutic purpose.

## Limitations

First, our study relied on previous reports, morphological and biochemical characteristics of the isolates for identification and confirmation.

Secondly, the MICs of the extracts were in milligram quantities signifying low potency compared to conventional antibiotics used in orthodox medicine.

Thirdly, because those plants were used by traditional doctors in diarrhoea cases, we did not bother conducting toxicity test to verify their level of safely.

These limitations not-withstanding, this study offers scientific information on why there should be evidence to justify any therapeutic combinations before embarking on it. Even herbs should not be combined arbitrarily.

## Supplementary information

**Additional file 1: Table S1.** The susceptibility pattern of the test isolates to conventional antibiotics. This data suggest the multi-drug resistant nature of the isolates used in the study.

## Data Availability

All the data needed in this work are provided in the manuscript and as additional file.

## Abbreviations

MIC: minimum inhibitory concentration
FIC: fractional inhibitory concentration
CF: methanolic *Cassytha filiformis* extract
PS: methanolic *Pterocarpus santalinoides* extract
AC: methanolic *Alchornea cordifolia* extract
GIT: gastrointestinal tract

## References

[1] Adonu CC, Esimone CO, Attama AA, Ugwueze MC (2013). In vitro evaluation of antibacterial activity of extracts from *Cassytha filiformis* linn against urogenital clinical gram-negative bacteria. Int J Pharm Biol Sci..

[2] Adonu CC, Enwa FO, Anie CO, Gugu T, Esimone CO, Attama AA (2013). *In vitro* evaluation of the combined effects of methanol extracts from *Cassytha filiformis* and *Cleistopholis patens* against *Pseudomonas aeruginosa* and *Escherichia coli*. Int J Adv Res.

[3] Ihedioha TE, Okechukwu VN, Ihedioha JI (2018). Effects of aqueous leaf infusion of *Pterocarpus santalinoides* DC on the serum lipid profile of guinea pigs (*Carvia porcellus*). J Complement Med Res.

[4] Lifongo LL, Simoben CV, Ntie-Kang F, Babiaka SB, Judson PN (2014). A bioactivity versus ethnobotanical survey of medicinal plants from Nigeria, West Africa. Nat Prod Bioprospect.

[5] Mythili S, Gajalakshmi S, Sathiavelu A, Sridharan TB (2011). Pharmacological activities of cassytha filiformis: a review. Asian J Plant Sci Res.

[6] Ogbonna PC, Idumah MC (2018). Phytochemical and mineral content in leaves, stem and bark of *Pterocarpus Santalinoides* (Nturukpa) from Afikpo, Ebonyi State, Nigeria. J Appl Sci Environ Manage.

[7] Okoye TC, Akah PA, Okoli CO, Ndu OO, Ezike AC, Okoye MO, Mbaoji FN, Agba EU (2010). Anti-diarrhoeal and antispasmodic effects of leaf extract of *Pterocarpus santalinoides*. Niger J Pharm Res.

[8] Njume C, Goduka NI (2012). Treatment of diarrhoea in rural african communities: an overview of measures to maximise the medicinal potentials of indigenous plants. Int J Environ Res Public Health.

[9] Okoye EL, Uba BO, Uhobo PC, Oli AN, Ikegbunam MN (2014). Evaluation of the antibacterial activity of methanol and chloroform extracts of *Alchornea cordifolia* leaves. J Scientific Res Rep.

[10] WHO. Diarrhoeal disease: facts sheet; 2017. https://www.who.int/news-room/fact-sheets/detail/diarrhoeal-disease. Accessed 15 May 2019.

[11] Thiam S, Diène AN, Fuhrimann S, Winkler MS, Sy I, Ndione JA (2017). Prevalence of diarrhoea and risk factors among children under five years old in Mbour, Senegal: a cross-sectional study. Infect Dis Poverty.

[12] Moshabela M, MacPherson P, Ezard N, Frean E, Mashimbye L, Elliott JH, Oldenburg B (2012). Clinical and social determinants of diarrhoeal disease in a rural HIV/AIDS clinic, South Africa: a case-control study. Int J STD AIDS.

[13] Keusch GT, Fontaine O, Bhargava A, et al. Diarrheal diseases. In: Jamison DT, Breman JG, Measham AR, et al., editors. Disease control priorities in developing countries, 2nd edn. Washington (DC): The International Bank for Reconstruction and Development/The World Bank; 2006. Chapter 19. https://www.ncbi.nlm.nih.gov/books/NBK11764/. Co-published by Oxford University Press, New York. 21250309

[14] Liu L, Oza S, Hogan D, Chu Y, Perin J, Zhu J, Lawn JE, Cousens S, Mathers C, Black RE (2016). Global, regional, and national causes of under-5 mortality in 2000-15: an updated systematic analysis with implications for the Sustainable Development Goals. Lancet.

[15] Colombara DV, Khalil IA-M, Rao PC (2016). Chronic health consequences of acute enteric infections in the developing world. Am J Gastroenterol Suppl..

[16] Lamberti LM, Fischer Walker CL, Black RE (2012). Systematic review of diarrhea duration and severity in children and adults in low- and middle-income countries. BMC Public Health.

[17] Argudín MÁ, Mendoza MC, Rodicio MR (2010). Food poisoning and *Staphylococcus aureus* enterotoxins. Toxins.

[18] Pinchuk IV, Beswick EJ, Reyes VE (2010). *Staphylococcal* enterotoxins. Toxins.

[19] Cheesman MJ, Ilanko A, Blonk B, Cock IE (2017). Developing new antimicrobial therapies: are synergistic combinations of plant extracts/compounds with conventional antibiotics the solution?. Pharmacogn Rev.

[20] Yuan H, Ma Q, Ye L, Piao G (2016). The traditional medicine and modern medicine from natural products. Molecules.

[21] Palombo EA (2011). Traditional medicinal plant extracts and natural products with activity against oral bacteria: potential application in the prevention and treatment of oral diseases. Evid Based Complement Altern Medicine eCAM.

[22] Falodun A. (2010). Herbal Medicine in Africa-Distribution, Standardization and Prospects. Research Journal of Phytochemistry.

[23] Komuro Akihiko (2017). Kampo Medicines for Infectious Diseases. Japanese Kampo Medicines for the Treatment of Common Diseases: Focus on Inflammation.

[24] Salami OO, Georgia CA (2013). The Assessment of the antimicrobial activities of *Ocimum gratissimum* (wild basil) and *Vernonia amygdalina* (bitter leaf) on some enteric pathogen causing dysentry or diarrhoea in patients. Int J Eng Sci.

[25] Basri DF, Fan SH (2005). The potential of aqueous and acetone extracts of galls of *Quercus infectoria* as antibacterial agents. Indian J Pharmacol.

[26] Cheesbrough M. District laboratory practice in tropical countries, 2nd Edn. Cambridge: Cambridge University Press. ISBN-13: 9781139449298.

[27] Ghamba PE, Balla H, Goje LJ, Halida A, Dauda MD (2014). *In*-*vitro* antimicrobial activities of *Vernonia amygdalina* on selected clinical isolates. Int J Curr Microbiol Appl Sci.

[28] Aiyegoro OA, Afolayan AJ, Okoh AI (2009). Synergistic interaction of *Helichrysum pedunculatum* leaf extracts with antibiotics against wound infection associated bacteria. Biol Res.

[29] Usha PTA, Jose S, Nisha AR (2010). Antimicrobial drug resistance—a global concern. Vet World.

[30] Wachtel-Galor S, Benzie IFF, Benzie IFF, Wachtel-Galor S (2011). Chapter 1. Herbal medicine: an introduction to its history, usage, regulation, current trends, and research needs. Herbal medicine: biomolecular and clinical aspects.

[31] Sofowora A, Ogunbodede E, Onayade A (2013). The role and place of medicinal plants in the strategies for disease prevention. Afr J Trad Complement Altern Med AJTCAM.

[32] Umeh SO, Umerie SC, Emelugo BN, Nwobi SC (2014). Preliminary study of the antibacterial and analgesic effect of the leaf extract of *Pterocarpus santalinoides* L’Hér. Ex DC. Int J Pharm Sci Invent.

[33] Kerantzas CA, Jacobs WA (2017). Origins of combination therapy for tuberculosis: lessons for future antimicrobial development and application. mBio.

[34] de Lera AR, Ganesan A (2016). Epigenetic polypharmacology: from combination therapy to multitargeted drugs. Clin Epigenet.

[35] Mohammadi Z, Jafarzadeh H, Shalavi S, Yaripour S, Sharifi F, Kinoshita JI (2018). a review on triple antibiotic paste as a suitable material used in regenerative endodontics. Iran Endod J.

[36] Enemchukwu CM, Oli AN, Okoye EI, Ujam NT, Osazuwa EO, Emechebe GO, Okeke KN, Ifezulike CC, Ejiofor OS, Okoyeh JN (2019). Winning the war against multi-drug resistant diarrhoeagenic bacteria. Microorganisms..

[37] Abdel-Aziz SM, Aeron A, Kahil TA, Garg N, Abdel-Aziz S, Aeron A (2016). Health benefits and possible risks of herbal medicine. Microbes in food and health.

[38] Parasuraman S, Thing GS, Dhanaraj SA (2014). Polyherbal formulation: concept of ayurveda. Pharmacogn Rev.

[39] Ujam NT, Oli AN, Ikegbunam MN, Adikwu MU, Esimone CO (2013). Antimicrobial resistance evaluation of organisms isolated from liquid herbal products manufactured and marketed in South Eastern Nigeria. Br J Pharm Res.

[40] Tachjian A, Maria V, Jahangir A (2010). Use of herbal products and potential interactions in patients with cardiovascular diseases. J Am Coll Cardiol.

[41] Ben-Arye E, Samuels N, Goldstein LH, Mutafoglu K, Omran S, Schiff E, Charalambous H, Dweikat T, Ghrayeb I, Bar-Sela G, Turker I, Hassan A, Hassan E, Saad B, Nimri O, Kebudi R, Silbermann M (2016). Potential risks associated with traditional herbal medicine use in cancer care: a study of Middle Eastern oncology health care professionals. Cancer..

[42] Adje DU, Oli AN (2013). Community Pharmacy in Warri, Nigeria—a survey of practice details. Sch Acad J Pharm.

