# Supplementary material for: Combinations of Alchornea cordifolia, Cassytha filiformis and Pterocarpus santalinoides in diarrhoegenic bacterial infections
Source: BMC Res Notes. 2019 Oct 7;12:649. doi: 10.1186/s13104-019-4687-0 (PMC6781413; doi:10.1186/s13104-019-4687-0)
Supplement: Supplementary file 1 — Additional file 1: Table S1. The susceptibility pattern of the test isolates to conventional antibiotics. This data suggest the multi-drug resistant nature of the isolates used in the study. [file 13104_2019_4687_MOESM1_ESM.docx]

**The susceptibility pattern of the test isolates to conventional antibiotics.**

| **Antibiotics** | ***Staphylococcus aureus*** | ***Salmonella typhi*** | ***Shigellae dysenteriae*** | ***Escherichia coli*** |
| --- | --- | --- | --- | --- |
| **Ceftaxidime (30µg)** | S | R | R | R |
| **Cefuroxime (30µg)** | S | R | S | R |
| **Gentamycin (10µg)** | S | S | R | R |
| **Cefixime (5µg)** | R | R | S | R |
| **Ofloxacin (5µg)** | S | S | R | S |
| **Augmentin (30µg)** | R | R | S | S |
| **Nitrofurantion (5µg)** | S | S | S | S |
| **Ciprofloxacin (5µg)** | S | S | S | S |

R = Resistance S = Susceptible
